# Non-IgE-mediated food hypersensitivity

**DOI:** 10.1186/s13223-018-0285-2

**Published:** 2018-09-12

**Authors:** Lori Connors, Andrew O’Keefe, Lana Rosenfield, Harold Kim

**Affiliations:** 1 Dalhousie University, Halifax, NS Canada; 2 Memorial University, St. John’s, NL Canada; 3 University of Manitoba, Winnipeg, MB Canada; 4 Western University, London, ON Canada; 5 McMaster University, Hamilton, ON Canada

## Abstract

Non-immunoglobulin E (IgE)-mediated food hypersensitivity includes a spectrum of disorders that predominantly affect the gastrointestinal tract. This review will focus on the following more common non-IgE-mediated food hypersensitivity syndromes: food protein-induced enterocolitis syndrome (FPIES), allergic proctocolitis (AP), food protein-induced enteropathy (FPE) and celiac disease. FPIES, AP and FPE typically present in infancy and are most commonly triggered by cow’s milk protein or soy. The usual presenting features are profuse emesis and dehydration in FPIES; blood-streaked and mucousy stools in AP; and protracted diarrhea with malabsorption in FPE. Since there are no confirmatory noninvasive diagnostic tests for most of these disorders, the diagnosis is based on a convincing history and resolution of symptoms with food avoidance. The mainstay of management for FPIES, AP and FPE is avoidance of the suspected inciting food, with periodic oral food challenges to assess for resolution, which generally occurs in the first few years of life. Celiac disease is an immune-mediated injury caused by the ingestion of gluten that leads to villous atrophy in the small intestine in genetically susceptible individuals. Serologic tests and small intestinal biopsy are required to confirm the diagnosis of celiac disease, and management requires life-long adherence to a strict gluten-free diet.

## Background

**Fig. 1:**
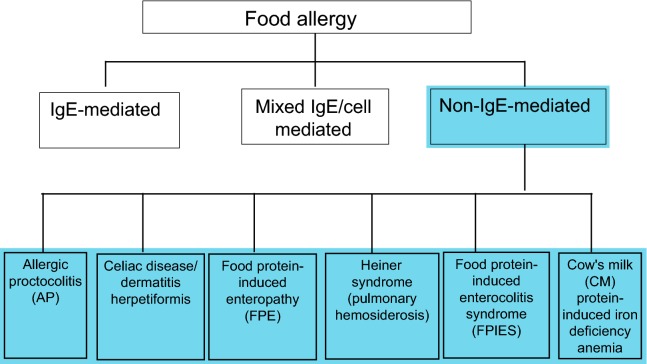
**Classification of non-IgE-mediated food hypersensitivity**

Non-immunoglobulin E (IgE)-mediated food hypersensitivity encompasses a wide spectrum of disorders including food protein-induced enterocolitis syndrome (FPIES), allergic proctocolitis (AP), food protein-induced enteropathy (FPE), celiac disease, Heiner syndrome (pulmonary hemosiderosis), and cow’s milk (CM) protein-induced iron deficiency anemia (see Fig. 1) [1–4]. Since Heiner syndrome and CM protein-induced iron deficiency anemia have become exceedingly rare, these non-IgE-mediated food hypersensitivities will not be discussed in this review.

Unlike IgE-mediated food allergy, the symptoms of non-IgE-mediated food hypersensitivity are typically delayed from hours to weeks after ingestion of the culprit food(s) [5]. Also, compared to IgE-mediated food allergies, the diagnosis of the various non-IgE-mediated food hypersensitivity syndromes can be challenging given the lack of noninvasive confirmatory tests for most of these disorders. Many of these non-IgE-mediated food hypersensitivity syndromes are diagnosed clinically based on history, and are managed empirically with food avoidance [6]. Therefore, it is important for physicians to be familiar with the key manifestations of these disorders and common offending foods. This review focuses on the classification, pathophysiology, epidemiology, clinical presentation, diagnosis and management of the more common non-IgE-mediated food hypersensitivity syndromes (for a review of IgE-mediated food allergy, please see article dedicated to this topic in this supplement).

## FPIES

Food protein-induced enterocolitis syndrome represents the more severe end of the non-IgE-mediated food hypersensitivity spectrum (Fig. 1). It usually occurs in young infants and generally affects the entire gastrointestinal tract, manifesting as profuse emesis, diarrhea and failure to thrive (Table 1) [3–5]. FPIES was first described in 1967 by Gryboski in an infant reacting to CM [7]. Although the pathophysiology of FPIES is not well understood, it has been postulated that food allergens may activate T cells in the intestinal epithelial lining, resulting in local inflammation, increased intestinal permeability, and fluid shifts [1]. However, the role of T cells has been questioned in several studies and further investigations are required to determine the exact mechanisms involved in the pathogenesis of this disorder [2].

**Table 1:** Key features of FPIES, AP and FPE [1–4]

	FPIES	AP	FPE
**Typical age of onset**	Days to 1 year; may be older in case of solid foods	Days to 6 months, usually 1–4 weeks; later onset in older children also reported	Dependent on age of exposure to antigen; CM and soy up to 2 years
**Food proteins implicated**			
Most common	CM, soy	CM, soy	CM, soy
Less common	Rice, oat, egg, barley, chicken, turkey, fish, pea	Wheat, egg, corn, meat, fish, sesame	Wheat, egg, soybean
**Symptoms**			
Emesis	Prominent	Absent	Intermittent
Diarrhea	Severe in chronic FPIES	Absent or mild	Moderate
Bloody stools	Severe in chronic FPIES	Prominent	Rare
Edema	Acute, severe	Mild, infrequent	Moderate
Shock	15–20%	Absent	Absent
Failure to thrive	Moderate in chronic FPIES	Absent	Moderate
Lethargy, pallor	Moderate	Absent	Absent
**Laboratory findings**			
Anemia	Moderate	Mild, infrequent	Moderate
Hypoalbuminemia	Acute	Mild, infrequent	Moderate
Malabsorption^b^	Absent	Absent	Present
Leukocytosis with neutrophils	Prominent	Absent	Absent
**Allergy evaluation**			
Food prick skin test	May be positive in 4–30%^a^	Negative	Negative
Serum food-allergen IgE	May be positive in 4–30%^a^	Negative	Negative
Total IgE	Normal or elevated	Normal or elevated	Normal
Peripheral blood eosinophilia	Absent	Occasional	Absent
**Biopsy findings**			
Villous injury	Patchy, variable	Absent	Variable, increased crypt length
Colitis	Prominent	Focal	Absent
Mucosal erosions	Occasional	Occasional, linear	Absent
Lymphoid nodular hyperplasia	Absent	Common	Absent
Eosinophils	Prominent	Prominent	Few
**OFC**	Vomiting, lethargy, pallor in 1–6 h; diarrhea in 5–8 h	Rectal bleeding in 6–72 h	Vomiting and/or diarrhea in 40–72 h

Adapted from Feuille and Nowak-Węgrzyn [1], Caubet et al. [2], Nowak-Wegrzyn [3], Nowak-Wegrzyn et al. [4]

*CM* cow’s milk, *FPIES* food protein-induced enterocolitis syndrome, *AP* allergic proctocolitis, *FPE* food protein-induced enteropathy, *IgE* immunoglobulin E, *OFC* oral food challenge

^a^If positive, might be a risk factor for persistent disease

^b^Malabsorption, steatorrhea, sugar malabsorption, and deficiency of vitamin K-dependent factors can be seen

The epidemiology of FPIES (as well as AP and FPE) has not been well studied. Cohort data suggest the incidence of CM-protein FPIES to be approximately 0.34% [8, 9].

Food protein-induced enterocolitis syndrome typically presents in the first 6–12 months of life with acute symptoms of severe emesis, diarrhea and dehydration that usually occur within 1–6 h after ingestion of the culprit food. Pallor, lethargy or hypotension/shock may also be present. The most common inciting allergens are CM protein and soy, although other triggers such as fish, egg, wheat and rice have been implicated [10, 11]. In the majority of children (65%), FPIES is caused by a single food (usually CM or soy); approximately 25% react to two foods while less than 10% react to three or more foods [11]. FPIES to CM and soy usually starts within the first 3–6 months of life, while FPIES to solid foods typically starts later, at 4–8 months of age, reflecting the sequence of introduction of these foods to the diet [3].

Less frequently, FPIES presents with chronic symptoms from ongoing exposure to the inciting allergen. Chronic FPIES, which has been described exclusively in infancy, is generally characterized by intermittent but progressive emesis and watery diarrhea with mucus and possibly blood [12]. It often leads to failure to thrive, hypoalbuminemia, metabolic derangements, and ultimately severe dehydration. There appears to be no clear temporal association between trigger exposure and the onset of symptoms. Symptoms usually resolve within a few days to 2 weeks after elimination of the culprit food.

## AP

Allergic proctocolitis (also referred to as allergic eosinophilic proctocolitis, dietary protein-induced proctocolitis, food protein-induced allergic proctocolitis, dietary protein-induced proctitis/proctocolitis, dietary protein-induced colitis, breast-milk-induced proctocolitis, eosinophilic proctitis, and benign dietary protein proctitis) represents the milder end of the non-IgE-mediated food hypersensitivity spectrum (Fig. 1). It typically presents in infants who seem generally healthy but who have visible specks or streaks of blood mixed with mucus in the stool (Table 1) [2, 5, 13]. These symptoms typically resolve with dietary avoidance, but recur on oral food challenge (OFC).

Allergic proctocolitis predominantly affects the rectosigmoid [3]. Although the exact mechanisms of AP are unknown, it is believed to result from maternal ingestion of a protein allergen (usually CM) which is passed through breast milk in a form that can be immunologically recognized [3]. It has also been suggested that AP is an antigen-induced colitis [14].

Allergic proctocolitis is thought to be a common cause of rectal bleeding in infancy, with prevalence estimates ranging widely from 0.16% to 64% of infants with isolated rectal bleeding [2, 15]. AP appears to be particularly common in breastfed infants, who account for approximately 60% of cases in published reports [3]. It also appears to be more common in countries with a lower prevalence of food allergy. A positive family history of atopy is present in up to 25% of infants with AP, and between 40% and 70% of infants with FPIES [4].

As mentioned earlier, AP is characterized by blood-streaked and mucousy normal to moderately loose stools in otherwise healthy, thriving infants. These characteristic symptoms can present within days of birth to 6 months of age, although older presentations have been noted [2, 3, 16, 17]. Also, there have been cases of the development of AP in children aged 2–14 years [18]. Some infants with AP may experience increased gas, episodic emesis, pain with defecation, and abdominal pain [3]. The most common causative foods in breast-fed infants with AP are CM, soy, egg and corn in the maternal diet, although other inciting foods such as meat, fish, apple, carrot, wheat, and sesame have been described [16]. AP in formula-fed infants is generally caused by CM and soy; extensively hydrolyzed formulas cause AP in up to 10% of cases [3].

### FPE

Food protein-induced enteropathy (also sometimes referred to as cow’s milk-sensitive enteropathy) is an uncommon syndrome of small-bowel injury with resulting malabsorption similar to that seen in celiac disease, although less severe [1, 4, 19] (Table 1). It is characterized by abnormal small intestinal mucosa while CM is in the diet, which is reversed by CM avoidance [19]. Eosinophils, CM-specific T-helper 2 lymphocytes, and localized production of IgE in the mucosa of the small intestine have been implicated in the pathophysiology of FPE [2]. Although the overall prevalence of FPE is unknown, reports suggest that the prevalence of this non-IgE-mediated food hypersensitivity syndrome has been declining over the last few decades [1].

Food protein-induced enteropathy presents with protracted diarrhea in the first 9 months of life (typically the first 1–2 months), and usually within weeks after the introduction of CM formula [2, 5]. Other food proteins, such as soybean, wheat, and egg, have also been implicated in FPE. More than half of affected infants also present with vomiting and failure to thrive, and some present with abdominal distension and early satiety [2, 5]. Bloody stools, however, are usually absent.

### Celiac disease

Celiac disease is an immune-mediated injury caused by the ingestion of gluten (a family of proteins found in grains such as wheat, rye and barley) in genetically susceptible individuals that leads to villous atrophy in the small intestine [20]. Dermatitis herpetiformis (also referred to as “celiac disease of the skin") is the chronic skin manifestation associated with celiac disease. It is classically described as clusters of vesicles on the extensor surfaces (“blisters”) that are intensely pruritic.

Genetic predisposition plays a key role in celiac disease. It is well known that the disease is strongly associated with specific human leukocyte antigen (HLA) class II genes known as *HLA*-*DQ2* and *HLA*-*DQ8.* Over 90% of affected patients have HLA-DQ2, and the remaining carry HLA-DQ8. With exposure to gluten, the immune system in affected individuals develops an inappropriate adaptive immune response. Gliadin interacts with intestinal cells to disassemble intra-epithelial tight junctions. Gliadin peptides can then pass through the epithelial barrier and activate CD4+ lymphocytes in the lamina propria. Inflammatory cytokines are then produced, leading to clonal expansion of B lymphocytes that differentiate into plasma cells that produce anti-tissue transglutaminase (anti-TTG) and anti-gliadin antibodies. The end result of this inflammatory cascade is villous atrophy and crypt hyperplasia seen on intestinal biopsy [21, 22].

Compared to the other non-IgE-mediated food allergies, the prevalence of celiac disease has been well studied. In Canada, celiac disease is estimated to affect 1% of the population [20], and the prevalence appears to be rising.

Celiac disease can manifest at any age once foods containing gluten are introduced into the diet. The classic symptoms of the disease include diarrhea, weight loss, and abdominal pain. However, symptomatology can be quite variable, including a myriad of intestinal and non-intestinal symptoms (see Table 2) [20]. Complications associated with celiac disease may include: malabsorption, osteoporosis/osteopenia, short stature, infertility and delayed puberty.

**Table 2:** Symptoms of celiac disease and associated conditions [20]

**Classic symptoms** • Abdominal distension • Abdominal pain • Chronic diarrhea • Anorexia • Irritability • Weight loss (or failure to thrive in children) • Muscle wasting • Dermatitis herpetiformis	**Non-classic symptoms** • Unexplained iron or folate deficiency anemia • Aphthous stomatitis (oral canker sores) • Dental enamel defects • Persistent/recurrent vomiting • Irritable bowel syndrome • Chronic constipation • Abnormal liver enzymes (ALT/AST) • Arthritis, arthralgia • Osteoporosis/osteopenia • Short stature • Delayed puberty • Infertility
**Associated conditions (% affected)** • Relative of individual with celiac disease (8–15%) • Type 1 diabetes mellitus (4–8%) • Autoimmune thyroiditis (2–5%) • Trisomy-21 (Down syndrome) (2–5%) • Turner syndrome (2–5%) • IgA deficiency (2–5%, up to 30% in patients with gastrointestinal symptoms)	**Neurological presentations** • Unexplained ataxia • Peripheral neuropathy • Epilepsy with occipital calcifications • Depression, anxiety • Fatigue

*IgA* immunoglobulin A, *ALT* alanine transaminase, *AST* aspartate transaminase

## Diagnosis

### FPIES, AP and FPE

Given the lack of specific diagnostic tests for FPIES, AP and FPE, the diagnosis of these disorders generally relies on a detailed medical history, physical examination, response to a trial of elimination of the suspected food (elimination diet) and OFCs [1–5, 13]. Diagnostic criteria for these disorders have been proposed and are summarized in Table 3 [2]. It is important to note that the differential diagnosis of FPIES, AP and FPE is broad and may include other allergic food disorders or gastrointestinal disorders, infectious diseases, mechanical or functional obstruction of the intestines, and metabolic, neurologic, and cardiac diseases.

**Table 3:** Proposed diagnostic criteria for FPIES, AP and FPE [2]

FPIES^a^	AP^b^	FPE^b^
1. < 2 years of age at first presentation (frequent but not mandatory) 2. Exposure to inciting food elicits repetitive and projectile vomiting, pallor, lethargy within 2–4 h; symptoms last a few hours, usually resolve within 6 h; diarrhea may be present, much less frequently and later (5–10 h) 3. Absence of symptoms that may suggest an IgE-mediated reaction 4. Avoidance of offending protein from the diet results in resolution of symptoms 5. Re-exposure or OFC elicits typical symptoms within 2–4 h; two typical episodes are needed to establish the definitive diagnosis without the need to perform an OFC	1. Small amount of rectal bleeding in an otherwise healthy infant 2. Disappearance of symptoms after all antigens are removed from diet 3. Exclusion of other cause of rectal bleeding	1. < 9 months of age at initial diagnosis 2. Repeated exposure to causative food elicits GI symptoms without alternative cause, predominantly vomiting and failure to thrive 3. Confirmation of the diagnosis by small bowel biopsy in a symptomatic child, showing villous injury, crypts hyperplasia, and inflammation 4. Removal of causative food results in resolution of symptoms within several weeks, although complete healing of villous injury may take several months

Adapted from Caubet et al. [2]

*FPIES* food protein-induced enterocolitis syndrome, *AP* allergic proctocolitis, *FPE* food protein-induced enteropathy, *IgE* immunoglobulin E, *GI* gastrointestinal, *OFC* oral food challenge

^a^Modified Powell’s diagnostic criteria

^b^There are no defined diagnostic criteria in the literature. These are criteria generally used to diagnose AP or FPE in clinical practice

#### Medical history

The evaluation of a patient with suspected food allergy or hypersensitivity begins with obtaining a thorough clinical history that considers the symptoms and clinical presentation (see previous section), potential inciting foods (notably CM, soy, fish, shellfish, eggs, nuts, and wheat), the temporal relationship between food ingestion and the onset of symptoms, as well as the clinical reproducibility of symptoms.

#### Physical examination

The physical examination should include thorough assessment of the gastrointestinal tract, as well as the respiratory tract and skin for supporting evidence of atopy and other allergic diseases, and to rule out the presence of other conditions that may mimic food allergy. In AP, the abdominal examination is usually normal and the infant appears generally well, although mild edema may be noted in some cases. Exclusion of other causes of rectal bleeding, such as infection, necrotizing enterocolitis, intussusception, or anal fissure, is essential [3]. In addition to the characteristic symptom of protracted diarrhea, infants with FPE may present with failure to thrive, abdominal distension, and moderate edema [2, 5].

#### Laboratory tests

The laboratory abnormalities noted in AP are usually mild and may include anemia, peripheral blood eosinophilia, hypoalbuminemia and hypoproteinemia (Table 1); elevated total serum IgE antibody levels may also be seen in some cases. In FPIES, moderate anemia may be noted, and leukocytosis with neutrophilia is prominent [1, 3, 4].

In FPE, malabsorption and moderate anemia are common (Table 1). Hypoproteinemia, steatorrhea, sugar malabsorption, and deficiency of vitamin K-dependent factors may also be observed. Although bloody stools are usually absent, occult blood can be found in 5% of patients [5]. There is generally no evidence of peripheral blood eosinophilia or elevations in total IgE levels in patients with FPE.

Testing for food-specific IgE is not routinely recommended for patients with AP and FPE, unless there are associated allergic conditions, such as atopic dermatitis, or immediate allergic symptoms to food ingestion [4]. However, skin prick tests or serum measurement of food-specific IgE may be considered prior to OFCs in patients with FPIES since 4–30% of these patients have or will develop specific IgE to the inciting food over time [1–4]. The diagnostic value of patch tests is controversial and, due to the lack of validation studies, these tests are not recommended for the routine diagnosis of non-IgE-mediated food hypersensitivity [2].

#### Endoscopy and biopsy

Endoscopy and biopsy are necessary for the conclusive diagnosis of FPE; the diagnosis is confirmed by the presence of villous injury, crypt hyperplasia, and inflammation on small-bowel biopsy specimens [2–5]. Biopsy is usually not indicated in AP or FPIES unless there is diagnostic uncertainty. However, if biopsy is performed in patients with AP, eosinophilic infiltration in the lamina propria and epithelium is evident in the vast majority of patients [5].

#### Elimination diet

A trial elimination diet is part of the diagnostic criteria for FPIES, AP and FPE to determine whether gastrointestinal symptoms are responsive to dietary manipulation [2]. Elimination of the offending food generally results in significant improvement of emesis and diarrhea within a few hours in patients with acute FPIES, and within days in patients with chronic FPIES. In AP, resolution of visible blood in the stool is usually noted within a few days. In patients with FPE, symptoms usually resolve within 1–4 weeks of elimination of the culprit food, although mucosal repair with normalization of disaccharidase activity may take several months [1, 2, 4].

#### Oral food challenge (OFC)

The OFC remains the gold standard to confirm the diagnosis of FPIES, AP or FPE after the resolution of symptoms under an elimination diet. It is also used to assess whether tolerance to the culprit food has developed [1–4]. In AP and FPE, reintroduction of the suspected food after 4–8 weeks of elimination can usually be performed at home and documented with a symptom diary. In FPIES, a physician-supervised OFC in an appropriate monitored setting may be considered because of the potential for severe reactions and need for intravenous hydration.

### Celiac disease

In individuals with symptoms suggestive of celiac disease (see Table 2), screening serologic tests should be performed [20, 23]. The immunoglobulin A (IgA) tissue transglutaminase antibody (IgA-TTG) or endomysium antibody (IgA-EMA) tests are recommended for initial testing, and should be performed by experienced laboratories. At most Canadian laboratories, anti-TTG is the initial screening test for celiac disease. Since these tests are IgA based, they will be falsely negative in patients with IgA deficiency. Therefore, screening for selective IgA deficiency should be performed at the same time as these serologic tests.

**Fig. 2:**
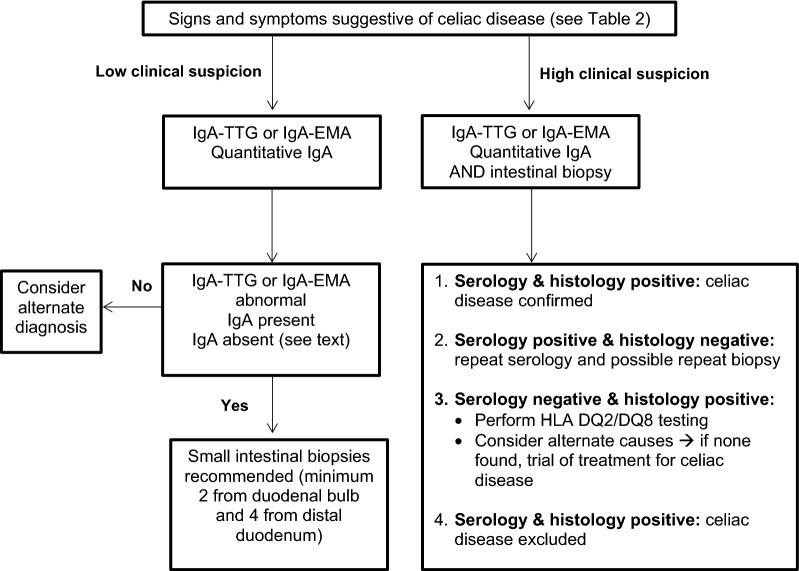
**Algorithm for the evaluation and diagnosis of celiac disease**. CD occurs in 2–5% of people with selective IgA deficiency. All symptomatic IgA deficient patients should be referred for endoscopic small intestinal biopsies regardless of their serology results, as false negatives can occur. In asymptomatic individuals with IgA deficiency, the laboratory may be able to perform IgG-TTG or an IgG-deamidated gliadin peptide (IgG-DGP). Negative HLA-DQ2 or DQ8 genetic tests are helpful to exclude the diagnosis of CD because over 99% of patients with CD are positive for HLA-DQ2 or DQ8. However, approximately 30% of the general population tests positive for one of these HLA types and most do not develop CD. *IgA* immunoglobulin A, *TTG* tissue transglutaminase antibody, *EMA* endomysium antibody, *HLA* human leukocyte antigens Adapted from Canadian Celiac Association Professional Advisory Council [20]

If screening is negative but clinical suspicion is high, a small intestinal biopsy (or skin biopsy in the case of dermatitis herpetiformis) should be performed to confirm the diagnosis. If screening is negative and clinical suspicion is low, an alternate diagnosis should be sought. If screening is positive and intestinal biopsy confirms the diagnosis, a gluten-free diet should be initiated. It is strongly recommended that both screening tests and biopsy be done *before* the patient is started on a gluten-free diet because eliminating gluten can interfere with making an accurate diagnosis. An algorithm for the evaluation of suspected celiac disease is provided in Fig. 2 [20].

## Management

### FPIES, AP and FPE

The cornerstone of the management of FPIES, AP and FPE is avoidance of the offending food(s). Referral to a dietitian and/or nutritionist can be very helpful in this regard, particularly for patients reacting to multiple foods.

For the acute management of FPIES reactions, rehydration may be required. Oral rehydration at home may be appropriate for mild reactions if fluids are tolerated by mouth. However, for severe emesis and lethargy, or if hypotension is present, then intravenous hydration in a medical setting will be essential [1]. Ondansetron may also be considered to control moderate to severe emesis. With rehydration and food avoidance, acute FPIES generally resolves in a few hours; patients with chronic FPIES usually return to good health in a few days to 2 weeks.

In breast-fed infants with AP, elimination of the offending food from the maternal diet (usually CM) generally leads to the resolution of gross bleeding within 72–96 h (although occult bleeding will take longer), and breast-feeding can be safely continued with continued avoidance of the culprit food protein [2, 3, 5]. However, in rare instances where symptoms are severe or when maternal avoidance of the offending trigger does not lead to symptom resolution, then a casein hydrolysate or an amino acid-based formula may be required [2, 3]. In patients with FPE, symptoms generally resolve within 1–4 weeks of trigger avoidance, although pathologic abnormalities may take up to 18 months to improve [5].

In formula-fed infants with these non-IgE-mediated food hypersensitivities to CM or to soy, guidelines recommend an extensively hydrolyzed formula as the first-line option, particularly in infants under 6 months of age with evidence of failure to thrive [24]. If this is not tolerated or if the patient’s initial inciting trigger is extensively hydrolyzed formula, then an amino acid formula is recommended. A study of infants with AP found significant improvements in physician-rated symptom scores, weight, and blood in the stool, as well as high parental satisfaction, with the use of an amino acid-based formula  [25]. A soy formula can be considered as an option for those with CM allergy who are 6 months of age or older without evidence of failure to thrive [24].

As mentioned earlier, periodic OFCs should be considered to determine whether the patient has developed tolerance to the food trigger. For both AP and FPE, foods can typically be reintroduced gradually at home if skin-prick tests and serum food-specific IgE antibody levels are negative, and if there is no history of a previous severe reaction [3, 4]. In FPIES, foods should be reintroduced under medical supervision due to the risk of hypotension. Delaying the introduction of higher risk foods may also be considered in the management of infants with FPIES, AP or FPE [1–4].

### Celiac disease

The treatment of celiac disease (including dermatitis herpetiformis) is lifelong adherence to a strict gluten-free diet [20, 23], and referral to a dietician with experience in celiac disease is encouraged for all patients. For dermatitis herpetiformis, dapsone may be required for symptom improvement.

## Prognosis

The prognosis of FPIES, AP, and FPE is generally good, with most affected individuals achieving tolerance in early childhood. In FPIES, overall remission rates range widely from 50 to 90% by the age of 6 years, and the timing of remission appears to be dependent on both the inciting food and the population studied [1]. In a study by Caubet and colleagues [11], the median age when tolerance was documented either by an OFC or by parental report of food reintroduction at home was 4.7 years for rice, 4 years for oat, 6.7 years for soy, and 5.1 years for CM in patients with undetectable milk-specific IgE. Another study found that, with the exception of soy, the median age of achieving tolerance to inciting foods was 24–28 months [26]. Mehr and colleagues [27] found that most subjects were tolerant to rice and soy by 3 years of age. It is important to note that infants with FPIES and concomitant IgE sensitization to the inciting trigger generally have a more protracted course and are at risk for the development of IgE-mediated food allergy [1]. For a more detailed review of IgE-mediated food allergy, please see article dedicated to this topic in this supplement.

Approximately half of patients with AP achieve tolerance by 1 year of age [17], and the vast majority by 3 years [3]. It has also been shown that up to 20% of breastfed infants with AP have spontaneous resolution of bleeding without changes in the maternal diet [16]. FPE usually resolves by age 1–2 years [4].

## Conclusions

Patients with FPIES, AP or FPE generally have a favourable prognosis, with the majority of cases resolving in the first few years of life. However, in some patients, the manifestations are severe, leading to shock in acute FPIES, or to failure to thrive in chronic FPIES or FPE. There is an urgent need to better characterize the pathophysiological mechanisms underlying these disorders in order to identify potential biomarkers for improved diagnosis as well as novel management strategies beyond food avoidance.

Celiac disease is common and the prevalence appears to be rising. Strict adherence to a gluten-free diet is the mainstay of therapy, which can be challenging for many patients. Therefore, novel therapies for the treatment of celiac disease are warranted.

## Key take-home messages

- FPIES, AP and FPE typically present in infancy and are most commonly triggered by CM protein or soy, although other food proteins such as rice, oat, egg, wheat, and fish have been implicated.
- AP represents the milder end of the non-IgE-mediated food hypersensitivity spectrum and is characterized by blood-streaked and mucousy stools in otherwise healthy infants.
- Acute FPIES presents with severe, projectile emesis, diarrhea, dehydration, and possibly shock. Chronic FPIES is less common and is generally characterized by intermittent but progressive emesis, watery diarrhea and failure to thrive.
- FPE is characterized by protracted diarrhea and malabsorption.
- The diagnosis of FPIES, AP and FPE generally relies on a careful and detailed medical history, physical examination, response to an elimination diet and OFCs. The diagnosis of FPE is confirmed by the presence of villous injury, crypt hyperplasia, and inflammation on small-bowel biopsy.
- The cornerstone of the management of FPIES, AP and FPE is avoidance of the offending food(s); symptoms generally resolve within days (for acute FPIES and AP) to weeks (for chronic FPIES or FPE) with trigger avoidance.
- Celiac disease is common and healthcare providers should maintain a high degree of suspicion for the disease since clinical manifestations can vary widely, including an array of intestinal and non-intestinal symptoms.
- The main therapeutic intervention for celiac disease is lifelong adherence to a gluten-free diet.

## Abbreviations

IgE: immunoglobulin E
FPIES: food protein-induced enterocolitis syndrome
AP: allergic proctocolitis
FPE: food protein-induced enteropathy
CM: cow’s milk
IgA: immunoglobulin A
HLA: human leukocyte antigen
TTG: tissue transglutaminase antibody
OFC: oral food challenge
